# Trajectories of labour market marginalisation among young adults with newly diagnosed attention-deficit/hyperactivity disorder (ADHD)

**DOI:** 10.1017/S2045796021000536

**Published:** 2021-10-22

**Authors:** M. Helgesson, S. Rahman, E. Björkenstam, K. Gustafsson, R. Amin, H. Taipale, A. Tanskanen, L. Ekselius, E. Mittendorfer-Rutz

**Affiliations:** 1 Division of Insurance Medicine, Department of Clinical Neuroscience, Karolinska Institutet, SE-17177 Stockholm, Sweden; 2 Department of Neuroscience, Psychiatry, Uppsala University, Uppsala, Sweden; 3 Department of Forensic Psychiatry, Niuvanniemi Hospital, Kuopio, Finland; 4 School of Pharmacy, University of Eastern Finland, Kuopio, Finland

**Keywords:** ADHD, attention-deficit/hyperactivity disorder, labour market marginalisation, sick leave

## Abstract

**Aims:**

Labour market marginalisation (LMM), i.e. severe problems in finding and keeping a job, is common among young adults with attention-deficit/hyperactivity disorder (ADHD). This study aimed to disentangle the extent of LMM as well as the heterogeneity in patterns of LMM among young adults with ADHD and what characterises those belonging to these distinct trajectories of LMM.

**Methods:**

This population-based register study investigated all 6287 young adults, aged 22–29 years, who had their first primary or secondary diagnosis of ADHD in Sweden between 2006 and 2011. Group-based trajectory (GBT) models were used to estimate trajectories of LMM, conceptualised as both unemployment and work disability, 3 years before and 5 years after the year of an incident diagnosis of ADHD. Odds ratios (ORs) with 95% confidence intervals (CIs) for the association between individual characteristics and the trajectory groups of LMM were estimated by multinomial logistic regression.

**Results:**

Six distinct trajectories of LMM were found: ‘increasing high’ (21% belonged to this trajectory group) with high levels of LMM throughout the study period, ‘rapidly increasing’ (19%), ‘moderately increasing’ (21%), ‘constant low’ (12%) with low levels of LMM throughout the study period, ‘moderately decreasing’ (14%) and finally ‘fluctuating’ (13%), following a reversed u-shaped curve. Individuals with the following characteristics had an increased probability of belonging to trajectory groups of increasing LMM: low educational level (moderately increasing: OR: 1.4; CI: 1.2–1.8, rapidly increasing: OR: 1.7; CI: 1.3–2.1, increasing high: OR: 2.9; CI: 2.3–3.6), single parents (moderately increasing: OR: 1.6; CI: 1.1–2.4, rapidly increasing: OR: 2.0; CI: 1.3–3.0), those born outside the European Union/the Nordic countries (rapidly increasing: OR: 1.7; CI: 1.1–2.5, increasing high: OR: 2.1; CI: 1.4–3.1), persons living in small cities/villages (moderately increasing: OR: 2.4; CI: 1.9–3.0, rapidly increasing: OR: 2.1; CI: 1.6–2.7, increasing high: OR: 2.6; CI: 2.0–3.3) and those with comorbid mental disorders, most pronounced regarding schizophrenia/psychoses (rapidly increasing: OR: 6.7; CI: 2.9–19.5, increasing high: OR: 12.8; CI: 5.5–37.0), autism spectrum disorders (rapidly increasing: OR: 4.6; CI: 3.1–7.1, increasing high: OR: 9.6; CI: 6.5–14.6), anxiety/stress-related disorders (moderately increasing: OR: 1.3; CI: 1.1–1.7, rapidly increasing: OR: 2.0; CI: 1.6–2.5, increasing high: OR: 1.8; CI: 1.5–2.3) and depression/bipolar disorder (moderately increasing: OR: 1.3; CI: 1.0–1.6, rapidly increasing: OR: 1.7; CI: 1.4–2.2, increasing high: OR: 1.5; CI: 1.2–1.9).

**Conclusions:**

About 61% of young adults were characterised by increasing LMM after a diagnosis of ADHD. To avoid marginalisation, attention should especially be given to young adults diagnosed with ADHD with a low educational level, that are single parents and who are living outside big cities. Also, young adults with comorbid mental disorders should be monitored for LMM early in working life.

## Background

The number of individuals diagnosed with attention-deficit/hyperactivity disorder (ADHD) in adult age has increased during the 2000s (Giacobini *et al*., 2018). Labour market marginalisation (LMM), that is severe problems in finding and keeping a job, is therefore common and has been reported to be widespread among patients with ADHD (Halmoy *et al*., 2009; Kupper *et al*., 2012). The reason is that attention deficiencies, lack of impulse control and problems with controlling the activity level may severely affect the ability to work (Wiklund *et al*., 2016; Helgesson *et al*., 2017; Hirvikoski *et al*., 2017). During the 2000s, the incidence of ADHD in young adults has increased considerably, which has led to public health concerns about the future health status as well as social and occupational development (Thomas *et al*., 2015; Edvinsson, 2017; Giacobini *et al*., 2018; Rydell *et al*., 2018). Still, today, the scientific knowledge on several aspects of LMM in young adults with ADHD is limited.

As the awareness of adult ADHD has increased, different treatment strategies have been introduced (Geffen and Forster, 2018; Lopez *et al*., 2018). Multimodal treatment, where the life situation of the person with ADHD is elucidated from a holistic perspective, is the primary recommendation from the Swedish National Board of Health and Welfare (National Board of Health and Welfare, 2014). The time of diagnosis is the starting point for different interventions aimed to enhance the ability to lead an everyday life as well as to increase the possibility to find and keep a position on the labour market. Pharmacological treatment may be added when non-pharmacological treatments do not have adequate effects. Studies have reported beneficial effects on functional ability when taking these medications (Vidal-Estrada *et al*., 2012; National Board of Health and Welfare, 2014; Geffen and Forster, 2018; Lopez *et al*., 2018).

Patients with ADHD often suffer from comorbidities such as common mental disorders, autism spectrum disorders and substance use disorders, but also from somatic diseases like asthma and diabetes mellitus (Kupper *et al*., 2012; Chen *et al*., 2013; Aduen *et al*., 2018; Cortese *et al*., 2018). Comorbidities with other medical conditions are important factors for functional impairment among persons diagnosed with ADHD (Halmoy *et al*., 2009; Edvinsson *et al*., 2013; Edvinsson, 2017; Helgesson *et al*., 2017). Moreover, sociodemographic/socioeconomic characteristics might affect the probability of experiencing LMM. Men are reported to have up to twice as high prevalence of ADHD than women (Thomas *et al*., 2015; Giacobini *et al*., 2018). The symptomatic picture seems to be different as men more often have externalising problems while women suffer more from internalising problems (Gershon and Gershon, 2002). This difference in symptomatic picture might therefore be a factor for the ability to find and hold a job. Also, other sociodemographic factors such as educational level, family composition, type of living area and region of birth might be of interest for the association between ADHD and LMM (Lehti *et al*., 2016; Giacobini *et al*., 2018).

LMM can be defined in different ways. The most used definition is unemployment (McKee-Ryan *et al*., 2005; Paul and Moser, 2009). The adverse consequences of ADHD on LMM can, however, be considerably underestimated by only including unemployment. Many with mental disorders are granted disability pension already at young adult age (OECD, 2003, 2010; Helgesson *et al*., 2017). For this reason, also measures of work disability such as sickness absence and disability pension, which are based on medical assessments, should be included in the conceptualisation of LMM in psychiatric epidemiological studies (OECD, 2010; Helgesson *et al*., 2018). Therefore, this study used a broad definition of LMM to be comprehensive and to increase the comparability to other countries, as welfare systems do differ to a large extent.

Patients diagnosed with ADHD are assumed to form a heterogeneous group with different degrees of comorbid disorders and different work-related as well as sociodemographic characteristics (Virtanen *et al*., 2020). Variation in these characteristics may therefore lead to considerable differences in patterns of LMM in this group. For this reason, this study aimed to disentangle this heterogeneity in patterns of LMM and investigate what characterises those young adults with ADHD following these distinct trajectory groups of LMM, both before and after the incident diagnosis. These analyses are warranted to create individual treatment and rehabilitation recommendations among young adults with ADHD. The specific aims were to: (1) investigate different trajectory groups of LMM, measured before and after an incident diagnosis of ADHD in adult age as the sum of months with unemployment, sickness absence and/or disability pension among young adults, and (2) to study to which extent specific medical, sociodemographic and work-related characteristics were associated with those trajectory groups.

## Methods

### The Swedish social insurance regulations

Individuals who are 16 years and above can receive sickness benefit when ill if they have a previous income from work. The employer covers the first 14 days of the period except for the first qualifying day. The Social Insurance Agency has the responsibility for payment from day 15 and onwards and information on the first 14 days is therefore not available in the registers. Those aged between 19 and 29 years can receive time-restricted disability pension if the work ability is reduced or if compulsory education is not completed at age 19. Individuals from age 30 years and above can be granted disability pension if the work capacity is permanently reduced. Individuals from 16 years and above can be enrolled at the Swedish Public Employment Service and can receive income-related unemployment benefit if they have had previous income from work. From age 20, there is, however, a possibility to receive basic levels of unemployment benefit also without previous income from work. From age 19 and above, it is possible to receive all the benefits included in the measure of LMM in this study.

### Registers

Data were merged individually based on the de-identified personal number and information was available for each individual from 1st of January 2003 to 31st of December 2016 from the following five Swedish nation-wide registers: (1) longitudinal integration database for health insurance and labour market studies (LISA), hosted by Statistics Sweden: sociodemographic variables and unemployment; (2) microdata for analysis of social security (MIDAS) hosted by the Social Insurance Agency: sickness absence and disability pension; (3) National Patient Register (NPR): main and secondary diagnoses for ADHD and comorbid disorders during the year of CED 2006–2011; (4) Prescribed Drug Register (PDR): prescription for ADHD-medication and (5) Cause of Death Register: date of mortality. Databases 3−5 are hosted by the Swedish National Board of Health and Welfare.

### Study population

The study base consisted of 8420 young adults between 22 and 29 years of age who had their incident main or secondary diagnosis of ADHD from either inpatient or specialised outpatient healthcare between 2006 and 2011. The lower age limit was motivated as all the participants were eligible for all ingoing benefits of the measure LMM at least three years before their first diagnosis of ADHD in adult age. A diagnosis of ADHD was defined by the code F90 in the International Classification of Diseases, Version 10 (ICD-10). Those with a record of medication for ADHD (Anatomical Therapeutic Chemical Classification (ATC) codes: N06BA01-13 and C02AC01-02) before the year of diagnosis were excluded (*n* = 2133). The final study population consisted of 6287 individuals.

### Variables

#### Outcome measure

LMM was measured as the annual sum of net months with work disability and unemployment during three years before and five years after the incident diagnosis of ADHD (cohort entry date, CED).

#### Covariates

(I) Sociodemographic factors: sex, age, educational level, region of birth, family composition and type of living area measured on 31st of December in the year preceding CED (see Table 1 for categorisation) and (II) medical factors: inpatient or specialised outpatient healthcare due to: depression and bipolar disorders (ICD-10: F30–F34), anxiety and stress-related disorders (F40–F48), autism spectrum disorders (F84), substance use disorders (F10–F19), behavioural and emotional disorders (F91–F98), schizophrenia/psychoses (F20–F29), mental retardation/developmental disorders (F70–F83, F85–F89), other mental disorders (Other F), musculoskeletal diseases (M01–M99), asthma (J45), diabetes mellitus (E10–E11), neoplasms (C00–D48), cardiovascular diseases (I00–I99), accidents (S00–S99) and other somatic diseases (other codes except for full-term uncomplicated delivery (O.80) and factors influencing health status and contact with health services (Z00–99)); prescription for medication for ADHD during the year after the diagnosis of ADHD, i.e. ‘centrally acting sympathomimetics’ ATC codes N06BA01-13 and ‘imidazoline receptor agonists’ C02AC01 and C02AC02 and subtype of hyperactivity disorder (F90), i.e. ADHD, F90.0 and F90.0B), attention-deficit disorder (ADD, F90.0C), dysfunction of attention, motor control and perception (Damp, F90.0A) and unspecified type of hyperactive disorder (UNS, F90.0X).

**Table 1.** Characteristics at baseline for the 6287 individuals, 22–29 years, diagnosed with attention deficit/hyperactivity disorder (ADHD) in adult age between 2006 and 2011

	*N* (%)
*Sociodemographic factors*	
Sex^a^	
Men	3556 (56.6)
Women	2731 (43.4)
Age^a^	
22–24 years	2653 (42.2)
25–29 years	3634 (57.8)
Educational level^a^	
Low (0–9 years)^b^	2869 (45.6)
Medium (10–12 years)	2795 (44.5)
High (>12 years)	623 (9.9)
Family composition^a^	
Married/living together without child at home	112 (1.8)
Married/living together with child at home	623 (9.9)
Single without child at home	5078 (80.8)
Single with child at home	474 (7.5)
Type of living area^a^	
Big cities	1987 (31.6)
Medium cities	2442 (38.8)
Small cities/villages	1858 (29.6)
Region of birth	
Sweden	5736 (91.2)
Nordic countries	45 (0.7)
EU27	64 (1.0)
Other countries	442 (7.0)
Unemployment^c^	
No days	3759 (59.8)
1–180 days	1629 (25.9)
>180 days	899 (14.3)
Sickness absence^c^	
No days	5009 (79.7)
1–90 days	419 (6.7)
>90 days	859 (13.7)
*Medical factors*	
Mental comorbidities^d^	
Depression/Bipolar disorder	1632 (26.0)
Anxiety/Stress-related disorders	1979 (31.5)
Autism spectrum disorder	666 (10.6)
Substance use	1300 (20.7)
Behavioural/Emotional disorders	207 (3.3)
Schizophrenia/Psychoses	210 (3.3)
Mental retardation	214 (3.4)
Other mental disorders	1149 (18.3)
Somatic comorbidities^d^	
Musculoskeletal disorders	358 (5.7)
Asthma/Diabetes	127 (2.0)
Accidents	738 (11.7)
Other somatic disorders	2532 (40.3)
ADHD-medication^e^	
Yes	3258 (51.8)
No	3029 (48.2)
Subtype of hyperactivity disorder	
Attention deficit/hyperactivity disorder (ADHD)	4927 (78.4)
Attention deficit disorder (ADD)	615 (9.8)
Deficits in attention, motor control and perception (DAMP)	57 (0.9)
Unspecified	688 (10.9)

a Measured on 31st of December in the year preceding the diagnosis of ADHD between 2006 and 2011.

b Missing education is considered to be low educational level.

c Measured during the year preceding the first diagnosis of ADHD.

d Measured during the same year as the diagnosis of ADHD due to following diagnoses in the International Classification of Diseases version 10 (ICD-10): Depression and Bipolar disorder (ICD-10: F30–F34), Anxiety and stress-related disorders (ICD-10: F40–F48), Autism spectrum disorder (ICD-10: F84), Substance use (ICD-10: F10–F19 and ATC: N07B), Behavioural and Emotional disorders (ICD-10: F91–F98), Schizophrenia/Psychoses (ICD-10: F20–F29), Mental retardation (ICD-10: F70–F83, F85–F89), Other mental disorders (ICD-10: Other F), Musculoskeletal diseases (ICD-10: M01–M99), Asthma (ICD-10: J45), Diabetes Mellitus (ICD-10: E10–E11 and ATC: A10), Neoplasms (ICD-10: C00–D48), Cardiovascular diseases (ICD-10: I00–I99), Accidents (S00–S99) and Other somatic diseases (ICD-10: other codes except O.80 and Z00–99).

e Measured during the year after the diagnosis of ADHD.

### Statistical analyses

Group-based trajectory (GBT) models were used to calculate trajectory groups of LMM from 3 years before to 5 years after the year of the incident diagnosis of ADHD. According to the founders/developers of the GBT model, the least complicated model based on existing knowledge, regarding both the number of trajectory groups and polynomial orders (Four polynomial orders are possible: 0 = constant level, 1 = a linear function which may constantly increase or decrease, 2 = quadratic level, i.e. that LMM can increase/decrease and then again decrease/increase and 3 = allows for several decreases and increases.), is to be chosen (Jones *et al*., 2001). General assumptions in this study were: (1) many individuals do not have any LMM during the study period. Therefore, a zero inflated Poisson (zip) model was chosen, and one trajectory is assumed to be constant low (polynomial order 0) (Wang *et al*., 2017; Helgesson *et al*., 2018), (2) many young adults have high levels of LMM already when entering working age which are sustained throughout adulthood. Therefore, one trajectory group is assumed to be linear (polynomial order 1), and (3) all other trajectory groups were assumed to have the possibility to change direction once (polynomial order 2). The association between several covariates and the trajectory groups of LMM was examined by multinomial logistic regression yielding odds ratios (ORs) with 95% confidence intervals (CIs) and using the group ‘constant low’, the group with least LMM, as the reference category. Log likelihood *χ*^2^ test estimated the association between a covariate and a specific trajectory.

## Results

### Characteristics of young adults diagnosed with ADHD

More men (57%) than women were diagnosed with ADHD during the study period (Table 1). A higher proportion was diagnosed at age 25–29 years (58%) than at age 22–24 years. Most individuals diagnosed with ADHD had low (46%) or medium (45%) educational level. The absolute majority were singles with no children living at home (81%). The most common mental comorbidities were anxiety/stress-related disorder (32%), depression/bipolar disorder (26%) and substance use disorders (21%). Somatic diseases such as musculoskeletal diseases (6%), accidents (12%) and other somatic diseases (40%) were also common among persons diagnosed with ADHD (Table 1). Around half of the persons diagnosed with ADHD (52%) had a record of ADHD-medication dispensing during the year after the diagnosis of ADHD.

### Trajectory groups of LMM

**Fig. 1.**
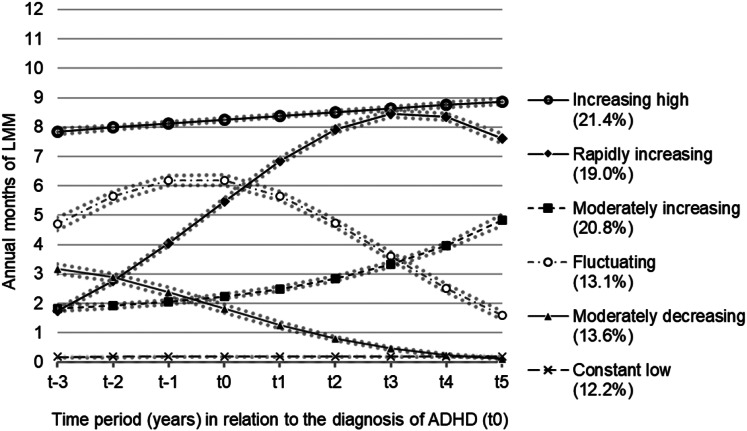
Trajectory groups of labour market marginalisation (LMM) among young adults diagnosed with attention-deficit hyperactivity disorder (ADHD, *n* = 6287).

Figure 1 shows the different trajectory groups of LMM of individuals with ADHD. Six distinct trajectory groups of LMM were established. Over 21% of all young adults followed the trajectory group of ‘increasing high’ LMM, starting from an average of eight annual months three years before the diagnosis and ending up with nine annual months five years after the diagnosis with ADHD. Another group, consisting of 19% of young adults with ADHD, followed a trajectory group of ‘rapidly increasing’ LMM, starting with just two annual months of LMM three years before the diagnosis, but ended up with eight annual months of LMM five years after the diagnosis. About 21% followed a trajectory group of ‘moderately increasing’ LMM, starting from two months of LMM at the beginning of the period and increasing to about five annual months of LMM at the end of the observation period. Moreover, around 12% belonged to the trajectory group of ‘constant low’ LMM and around 14% comprised the group of ‘moderately decreasing’ LMM, beginning with three annual months of LMM and decreasing to very low LMM levels at the end of the observation period. Finally, about 13% followed the trajectory group of ‘fluctuating’ LMM, starting with about five months three years before the diagnosis, with an increase to over six months of LMM in the year of the diagnosis, and then sharply declining to under two annual months five years after the diagnosis.

### Sociodemographic factors by trajectory group

Compared to men, women had higher odds of following the ‘increasing high’ trajectory group of LMM (OR: 1.5; CI: 1.2–1.9) compared to the ‘constant low’ trajectory group of LMM (Table 2). The odds of following the ‘increasing high’ (OR: 1.8; CI: 1.4–2.1) and ‘fluctuating’ (OR: 1.7; CI: 1.4–2.1) trajectory groups of LMM compared to the ‘constant low’ trajectory group of LMM were higher among persons aged 25–29 compared to persons aged 22–24. Persons with a low educational level had higher odds of belonging to all trajectory groups of increasing LMM (‘increasing high’ OR: 2.9; CI: 2.3–3.6, ‘rapidly increasing’ OR: 1.7; CI: 1.3–2.1, ‘moderate increasing’ OR: 1.4; CI: 1.2–1.8) but also the fluctuating trajectory group (OR: 1.5; CI: 1.2–1.9) compared to belonging to the ‘constant low’ trajectory group of LMM. Persons with high educational level had lower odds of following all other trajectory groups compared to ‘constant low’ LMM (OR range: 0.2–0.6; CI range: 0.1–0.7). Singles living with children at home had a higher odds of following trajectory groups of ‘rapidly increasing’ LMM (OR: 2.0; CI: 1.3–3.0) and ‘moderately increasing’ LMM (OR: 1.6; CI: 1.1–2.4). Persons living outside big cities, both in medium cities or in villages, had higher odds of following all other trajectory groups of LMM (OR range: 1.6–2.6; CI range: 1.2–3.3), compared to belonging to the ‘constant low’ trajectory group of LMM. Finally, individuals born outside the European Union/the Nordic countries had a higher probability of following trajectory groups of increased LMM (‘increasing high’ OR: 2.1; CI: 1.4–3.1; ‘rapidly increasing’ OR: 1.7; CI: 1.1–2.5).

**Table 2.** Adjusted^a^ odds ratios (OR) and 95% confidence intervals (CI) for belonging to each trajectory group of labour market marginalisation (LMM) compared to the reference group (constant low trajectory of LMM) among the individuals aged 22–29 years and registered in Sweden, with an incident diagnosis of attention-deficit hyperactivity disorder (ADHD) 2006–2011 (*n* = 6287)

	Moderately decreasing *v*. Constant low	Moderately increasing *v*. Constant low	Fluctuating *v*. Constant low	Rapidly increasing *v*. Constant low	Increasing high *v*. Constant low	
	OR (95% CI)	OR (95% CI)	OR (95% CI)	OR (95% CI)	OR (95% CI)	Log-likelihood test (*p*-value)^b^
*Sociodemographic factors*						
Sex						
Men	1	1	1	1	1	47.1 (<0.0001)
Women	0.81 (0.65–1.01)	0.93 (0.76–1.14)	0.85 (0.67–1.07)	1.03 (0.83–1.27)	**1.50** (**1.21–1.86)**^c^	
Age						
22 –24 years (younger)	1	1	1	1	1	118.9 (<0.0001)
25–29 years (older)	0.87 (0.71–1.07)	0.84 (0.69–1.01)	**1.70** (**1.37–2.11)**	0.97 (0.80–1.19)	**1.75** (**1.43–2.14)**	
Educational level						
Low (0–9 years)	1.19 (0.96–1.49)	**1.44** (**1.17–1.76)**	**1.49** (**1.19–1.87)**	**1.66** (**1.34–2.06)**	**2.88** (**2.32–3.58)**	294.2 (<0.0001)
Medium (>9–12 years)	1	1	1	1	1	
High (>12 years)	**0.39** (**0.28–0.53)**	**0.56** (**0.43–0.73)**	**0.34** (**0.24–0.47)**	**0.34** (**0.25–0.46)**	**0.20** (**0.14–0.28)**	
Family composition						
Married/living together without child at home	0.95 (0.42–2.13)	0.71 (0.33–1.54)	1.57 (0.77–3.30)	1.40 (0.70–2.89)	1.23 (0.61–2.58)	47.7 (<0.0001)
Married/ living together with child home	0.87 (0.62–1.21)	0.90 (0.67–1.21)	0.78 (0.55–1.09)	1.03 (0.75–1.40)	**0.52** (**0.37–0.73)**	
Single without child	1	1	1	1	1	
Single with child	1.22 (0.77–1.92)	**1.56** (**1.05–2.36)**	1.46 (0.94–2.29)	**1.95** (**1.31–2.95)**	1.08 (0.71–1.65)	
Type of living area						
Big cities	1	1	1	1	1	72.3 (<0.0001)
Medium cities	**1.71** (**1.36–2.15)**	**1.62** (**1.31–2.00)**	**1.55** (**1.22–1.96)**	**1.59** (**1.27–1.98)**	**1.95** (**1.55–2.44)**	
Small cities/villages	**2.09** (**1.60–2.72)**	**2.36** (**1.85–3.01)**	**2.08** (**1.59–2.73)**	**2.09** (**1.62–2.69)**	**2.57** (**1.98–3.33)**	
Region of birth						
Sweden	1	1	1	1	1	28.5 (0.0186)
Nordic countries	0.68 (0.19–2.34)	0.87 (0.31–2.64)	**0.13** (**0.01–0.81)**	0.85 (0.29–2.66)	1.14 (0.40–3.58)	
EU27	0.35 (0.09–1.05)	0.94 (0.42–2.14)	0.83 (0.31–2.13)	0.87 (0.37–2.11)	0.62 (0.24–1.58)	
Other countries	1.09 (0.70–1.69)	1.41 (0.97–2.10)	**1.66** (**1.10–2.53)**	**1.66** (**1.12–2.48)**	**2.08** (**1.41–3.10)**	
*Medical factors*						
Comorbidities^d^						
Depression/Bipolar disorder	**1.33** (**1.04–1.71)**	**1.28** (**1.02–1.61)**	**1.82** (**1.42–2.32)**	**1.74** (**1.38–2.19)**	**1.47** (**1.17–1.87)**	34.2 (<0.0001)
Anxiety/Stress-related disorders	1.24 (0.98–1.57)	**1.33** (**1.07–1.66)**	**1.70** (**1.35–2.16)**	**2.03** (**1.63–2.53)**	**1.84** (**1.47–2.30)**	59.1 (<0.0001)
Autism spectrum disorder	1.37 (0.85–2.24)	1.48 (0.96–2.32)	**2.39** (**1.53–3.79)**	**4.63** (**3.13–7.07)**	**9.61** (**6.53–14.58)**	292.8 (<0.0001)
Substance use	**1.64** (**1.26–2.16)**	**1.32** (**1.02–1.71)**	**1.35** (**1.02–1.79)**	**1.40** (**1.08–1.83)**	0.88 (0.67–1.16)	36.5 (<0.0001)
Behavioural/Emotional disorders	0.69 (0.32–1.48)	1.11 (0.60–2.14)	1.27 (0.65–2.55)	**1.91** (**1.07–3.61)**	**1.93** (**1.07–3.66)**	17.3 (0.0040)
Schizophrenia/Psychoses	1.21 (0.40–4.05)	1.91 (0.75–5.88)	2.40 (0.90–7.57)	**6.66** (**2.86–19.47)**	**12.75** (**5.54–37.02)**	104.8 (<0.0001)
Mental retardation	0.89 (0.41–1.94)	0.78 (0.39–1.64)	0.95 (0.43–2.12)	1.43 (0.75–2.88)	**3.94** (**2.21–7.60)**	76.4 (<0.0001)
Other mental disorders	1.09 (0.80–1.50)	1.24 (0.94–1.66)	**1.64** (**1.21–2.23)**	**2.04** (**1.55–2.72)**	**2.85** (**2.16–3.79)**	103.6 (<0.0001)
Musculoskeletal disorders	0.75 (0.46–1.21)	1.12 (0.75–1.69)	0.92 (0.57–1.47)	1.18 (0.78–1.81)	1.15 (0.75- 1.79)	6.1 (0.2981)
Asthma/Diabetes	1.25 (0.50–3.28)	1.26 (0.55–3.11)	**2.27** (**1.01–5.61)**	2.04 (0.95–4.93)	**2.31** (**1.08–5.55)**	9.0 (0.1103)
Accidents	1.12 (0.80–1.57)	**1.36** (**1.01–1.85)**	**1.50** (**1.07–2.10)**	1.26 (0.92–1.73)	1.03 (0.74–1.45)	11.0 (0.0506)
Other somatic disorders^e^	1.07 (0.87–1.33)	1.18 (0.97–1.44)	0.91 (0.73–1.14)	1.17 (0.95–1.43)	1.17 (0.95–1.44)	10.3 (0.0678)
ADHD-medication^f^						
No	1	1	1	1	1	31.9 (<0.0001)
Yes	1.10 (0.90–1.34)	1.00 (0.83–1.20)	0.89 (0.73–1.10)	0.97 (0.80–1.17)	**0.69** (**0.56–0.84)**	
Subtype of hyper-activity disorder						
ADHD	1	1	1	1	1	22.2 (0.1025)
Attention deficit disorder (ADD)	1.01 (0.70–1.45)	1.14 (0.83–1.58)	1.27 (0.89–1.82)	**1.43** (**1.03–2.00)**	1.32 (0.95–1.86)	
Deficits in attention, motor control and perception (DAMP)	2.33 (0.68–10.64)	1.66 (0.50–7.49)	0.30 (0.02–2.36)	2.69 (0.84–11.95)	2.94 (0.95–12.91)	
Unspecified	0.94 (0.68–1.31)	0.86 (0.64–1.17)	0.78 (0.55–1.10)	1.11 (0.82–1.51)	1.04 (0.77–1.42)	

a All ORs were mutually adjusted for all other variables.

b *χ*^2^ statistics from the log-likelihood test (*p*-value) derived by multinomial logistic regression.

c Significant associations are in bold text.

d Reference group regarding all comorbidities were those without the respective health condition.

e All somatic disorders except musculoskeletal diseases, asthma, diabetes, or accidents.

f Measured during the year after the diagnosis of ADHD.

### Medical factors

Especially high odds of belonging to the trajectory groups of ‘increasing high’ and/or ‘rapidly increasing’ LMM compared to those belonging to the trajectory groups of ‘constant low’ LMM were seen among those with comorbid schizophrenia/psychoses (‘increasing high’ OR: 12.8; CI: 5.5–37.0 ‘rapidly increasing’ OR: 6.7; CI: 2.9–19.5), autism spectrum disorders (‘increasing high’ OR: 9.6; CI: 6.5–14.6 ‘rapidly increasing’ OR: 4.6; CI: 3.1–7.1) and mental retardation (‘increasing high’ OR: 3.9; CI: 2.2–7.6, Table 2). High odds for ‘increasing high’ and/or ‘rapidly increasing’ LMM were also seen among those with comorbid anxiety/stress-related disorders (‘increasing high’ OR: 1.8; CI: 1.5–2.3 ‘rapidly increasing’ OR: 2.0; CI: 1.6–2.5), depression/bipolar disorder (‘increasing high’ OR: 1.5; CI: 1.2–1.9, ‘rapidly increasing’ OR: 1.7; CI: 1.4–2.2), asthma/diabetes (‘increasing high’ OR: 2.3; CI: 1.1–5.6) and behavioural/emotional disorders (‘increasing high’ OR: 1.9; CI: 1.1–3.7, ‘rapidly increasing’ OR: 1.9; CI: 1.1–3.6). Additionally, higher odds among individuals with comorbid disorders belonging to the ‘fluctuating’ trajectory were seen among those with comorbid autism spectrum disorders (OR: 2.4; CI: 1.5–3.8), asthma/diabetes (OR: 2.3; CI: 1.0–5.6), depression/bipolar disorder (OR: 1.8; CI: 1.4–2.3) and anxiety/stress-related disorders (OR: 1.7; CI: 1.4–2.2).

Those who utilised ADHD-medication during the year after the ADHD diagnosis had lower odds (OR: 0.7; CI: 0.6–0.8) of belonging to the ‘increasing high’ trajectory of LMM compared to the ‘constant low’ trajectory of LMM. Those diagnosed by ADD had higher risk of belonging to ‘rapidly increasing’ trajectory of LMM compared to the ‘constant low’ trajectory of LMM.

## Discussion

### Main findings

The results revealed that a majority (61%) of young adults diagnosed with ADHD in adult age had a poor prognosis regarding subsequent LMM following trajectory groups of increasing LMM on different levels during the study period. Young adults with comorbid mental disorders, most pronounced regarding schizophrenia/psychoses, autism spectrum disorders, depression/bipolar disorders, and anxiety/stress-related disorders had a high probability of following trajectory groups of increasing LMM during the study period. Also, those with a low educational level, persons with children living at home, single parents, and individuals living outside big cities had an increased probability of belonging to trajectory groups of increasing LMM.

#### Trajectory groups of LMM

Nearly two thirds of all young adults diagnosed with ADHD were found to have a poor prognosis regarding future LMM. Previous Swedish studies reported that the risk of having disability pension was high, about 10−15 times higher among individuals diagnosed with ADHD than among controls without ADHD (Virtanen *et al*., 2020; Helgesson *et al*., 2021). Furthermore, as we conceptualised LMM as both work disability and unemployment, our study shows that unemployment was an equally large driving force for future LMM among young adults diagnosed with ADHD. Previous studies on other diagnoses and subsequent LMM show a somewhat different picture. Among young adults with common mental disorders, 50% were found to have a poor prognosis for later LMM (Helgesson *et al*., 2018). In a comparison with young adults from the general population without any mental disorder, the risk of experiencing trajectories of increasing LMM was about 9% (Helgesson *et al*., 2018), as compared to 61% among young adults diagnosed with ADHD. A study on individuals (aged 19–60) with low back pain, just about 20% had a poor prognosis for later LMM (Dorner *et al*., 2018). Young adults diagnosed with ADHD had thus a relatively poor prognosis regarding later LMM, both compared to individuals with other health conditions, but foremost in comparison to the general population without mental disorders. One straightforward explanation might be that the symptoms of inattention and hyperactivity are hard to cope with at a workplace. During the last decades, a rapid increase in adult ADHD has been seen, and employers might still not have sufficient knowledge on how to adapt the work situation for those diagnosed with ADHD (Kupper *et al*., 2012; Thomas *et al*., 2015; Giacobini *et al*., 2018). Another reason might be that the recommendations and interventions stipulated by the Swedish National Board of Health and Welfare (National Board of Health and Welfare, 2014) are not sufficient, or are not used as intended. Only about 13% of all young adults with ADHD followed the ‘fluctuating’ trajectory group of LMM, indicating that the intervention at the time of the diagnosis had a good outcome. Optimally, many more would follow this trajectory. One reason for this result might be that many who are diagnosed with ADHD do suffer from severe comorbidities (Virtanen *et al*., 2020). Studies, however, show that the risk of LMM is also high when comorbidities are accounted for (Virtanen *et al*., 2020). As nearly two-thirds of all young adults diagnosed with ADHD do have a very volatile connection to the labour market, the recommendations and policies for work rehabilitation may need revision.

### Sociodemographic factors

Studies have shown sex differences with regard to symptoms of ADHD (Gershon and Gershon, 2002; Edvinsson *et al*., 2013) and women have generally higher levels of work disability compared to men (Allebeck and Mastekaasa, 2004). The assumption was therefore that there would be significant differences between men and women also regarding patterns of LMM. However, the differences between men and women were found to be rather modest, but noteworthy is that women to a higher extent followed the trajectory of ‘increasing high’ LMM compared to men. One explanation of these findings can be that women and men, on a group level, have been found to have a different symptomatic picture, the more internalising problems found among women may lead to that women to a less extent receive a diagnosis of ADHD, and hence women who are diagnosed with ADHD have more severe symptoms (Gershon and Gershon, 2002).

The group of young adults over 24 years seemed to have a higher probability of following the trajectory of ‘fluctuating’ LMM compared to the younger group. Also, those over 24 years followed, to a greater extent, the ‘increasing high’ trajectory group of LMM. These findings are in line with other studies which conclude that the risk of LMM increases with age (Karlsson *et al*., 2008). Young adults with low educational level who were diagnosed with ADHD had a high probability of belonging to trajectory groups of increasing LMM. One might think that the relatively low share of high educational level in the young population depends on their rather young age when having their diagnosis. In a study on individuals in the same age diagnosed with common mental disorders, the share of high educational level was 2.5 times higher than among young adults diagnosed with ADHD. According to the criteria for Diagnostic and Statistical Manual of Mental Disorders, 4th Edition (DSM-IV), childhood problems with inattention and hyperactivity before age 12 must be present in order to be diagnosed with ADHD in adulthood (APA, 2000). Those with most severe symptoms of ADHD may have struggled in attaining adequate education. An adequate education, at least upper secondary school, is of high importance for a positive attachment to the labour market and is congruent to the findings of other studies (Helgesson *et al*., 2017, 2018; Robroek *et al*., 2020).

Single parents, diagnosed with ADHD, had an increased probability for belonging to the trajectory groups of ‘moderately increasing’ and ‘rapidly increasing’ LMM. Mothers diagnosed with ADHD are reported to struggle more regarding parenting compared to mothers without ADHD (Murray and Johnston, 2006). This may lead to parental stress, and there does not seem to be any differences between male and female parents regarding this (Johnston *et al*., 2012; Waite and Ramsay, 2010). The parental stress may, in turn, lead to higher propensity to have depression, anxiety or stress-related disorders (Theule *et al*., 2011), disorders known to have a detrimental effect on work capacity (Helgesson *et al*., 2018). Moreover, single parents with mental disorders are, in general, more vulnerable in their role as parents (Theule *et al*., 2011). Support to single parents diagnosed with ADHD is therefore warranted to prevent persistent LMM.

The risk of LMM was higher among those diagnosed with ADHD, who are born outside the European Union/the Nordic countries. The general risk of high LMM among non-European migrants with mental disorders has been reported by other studies (Helgesson *et al*., 2017, 2019), and the reasons seem to be multifaceted. Often acknowledged reasons to differences in labour attachment between migrants and the host population is the generally lower educational level and worse health status among migrants. These factors were adjusted in the analyses, but there might be residual confounding related to those factors. Healthcare utilisation is generally lower among migrants (Lindert *et al*., 2008), and those who are diagnosed with ADHD might therefore have higher severity of the disease.

### Medical factors

Young adults diagnosed with ADHD who had mental comorbidities as depression/bipolar disorders, anxiety/stress-related disorders, autism spectrum disorders, and schizophrenia/psychoses had a high probability of belonging to trajectory groups of increasing LMM. The lifetime prevalence of comorbid mental disorders was also found to be very high, also confirmed by other studies (Jacob *et al*., 2007; Edvinsson *et al*., 2013). Studies have reported that mental disorders by themselves are related to a high risk of LMM (McEvilly *et al*., 2015; Helgesson *et al*., 2017, 2018; Virtanen *et al*., 2020). Many with comorbid mental disorders, however, belonged to the trajectory group of ‘fluctuating’ LMM, which indicates a good prognosis regarding future labour market attachment. This might be due to a good response to the interventions aiming for a better everyday life as well as a better working life, stipulated by the Swedish National Board of Health and Welfare (National Board of Health and Welfare, 2014). Some studies have reported that strategies and policies aiming to reintegrate persons with work disability due to mental disorders back to the labour market have been successful (Bejerholm *et al*., 2015; Kuznetsova and Yalcin, 2017). A review on occupational functioning in patients for ADHD concludes: ‘Importantly, ADHD is a treatable condition, and patients, employers and physicians have a role to play in ensuring optimal occupational health’ (Kupper *et al*., 2012).

Comorbid disorders are to some extent a consequence of the problems which persons with ADHD encounter in everyday life. High severity of ADHD symptoms seems to correlate with higher rates of both anxiety and depression (Michielsen *et al*., 2013). Therefore, support early in life may prevent the occurrence of disabling depression, anxiety- and stress-related disorders, thereby decreasing the probability for LMM in young adult age. Although many young adults with ADHD and comorbidities belonged to the trajectory groups of increasing LMM, there are also many that have a good prognosis of future attachment to the labour market. A review concludes that many with ADHD have an extraordinary work ability if only the prerequisites are beneficial (Kupper *et al*., 2012). To prevent long-term marginalisation of young adults with ADHD, more knowledge on which treatment options that are successful for integrating young adults into the labour market is warranted. Information and awareness programmes could also increase employers’ knowledge on the disorder and provide information on how to adapt working tasks to optimise work ability in this patient group (Kupper *et al*., 2012).

In this study, we found an indication that a record of ADHD-medication during the year after the diagnosis of ADHD decreased the risk of permanent high LMM. Also, other studies have reported slight improvements regarding the possibility to work when taking medication (Geffen and Forster, 2018; Giacobini *et al*., 2018). However, studies show that discontinuation of ADHD medication is common (Edvinsson and Ekselius, 2018). Individuals receiving ADHD medication may be a selected group and thus, studies with a design that are able to better control treatment bias are warranted before robust conclusions can be drawn of the effect of ADHD-medication for later LMM.

## Strengths and limitations

The main strengths of this study were the use of register data with high quality, including individual information on a vast number of sociodemographic and health-related covariates (Ludvigsson *et al*., 2011, 2016). Another strength was the population-based design including all young adults between 22 and 29 who were diagnosed with ADHD in adult age and a long follow-up period (nine years) with little loss to follow-up. Finally, the conceptualisation of LMM, including both work disability and unemployment reduces the risk of underestimating the true rate of marginalisation among young adults diagnosed with ADHD.

Limitations worth mentioning are that ADHD as well as all comorbid disorders were measured from visits to specialised healthcare. Hence, information on conditions treated in primary care has not been covered. Still, the number of young adults with ADHD might not have been severely affected, as those patients are mainly diagnosed and treated within specialised healthcare. Finally, data on sickness absence only includes information of spells longer than 14 days as the first two weeks are covered by the employers and information on unemployed individuals who are not registered by The Swedish Employment Agency is not covered by this study. This might slightly underestimate the risk of LMM among individuals with ADHD.

## Conclusions

Nearly two thirds of young adults diagnosed with ADHD in adult age are characterised by patterns of increasing LMM during the follow-up period. To avoid marginalisation, young adults with comorbid mental disorders must be monitored for LMM early in working life. Also, special attention must be given to young adults with low educational level, singles with children and those living in medium cities or villages.

## Data Availability

The data of this study are available from Statistics Sweden, The Swedish Social Insurance Agency and the Swedish National Board of Health and Welfare. The data are not publicly available but can be used if an ethical permission is obtained.
